# An Unusual Case of Acute Abdomen With Jaundice

**DOI:** 10.7759/cureus.76155

**Published:** 2024-12-21

**Authors:** John Bukasa Kakamba, Shruti Wadhwani, Ayrton I Bangolo, Aaron Ngandu, Ben Bepouka, Daddy Liombo Mbiso, Junior Koke Opanga, Jerry Nsimba Basolua, Serge Kakamba, Aurelien Siasia, Pascal M Bayauli, Simcha Weissman, Nikita Wadhwani, Jean René M’Buyamba-Kabangu

**Affiliations:** 1 Endocrinology, University Clinics of Kinshasa, Kinshasa, COD; 2 Endocrinology, University of Liege, Liege, BEL; 3 Internal Medicine, Hackensack Meridian Health Palisades Medical Center, North Bergen, USA; 4 Hematology and Oncology, John Theurer Cancer Center at Hackensack University Medical Center, North Bergen, USA; 5 Public Health, University of Kinshasa, Kinshasa, COD; 6 Infectious Diseases, University Clinics of Kinshasa, Kinshasa, COD; 7 Radiology, University Clinics of Kinshasa, Kinshasa, COD; 8 Internal Medicine, Rapha Clinic, Kinshasa, COD; 9 Internal Medicine, Livulu Center For Physically Disabled, Kinshasa, COD; 10 Urology, Ibn Rochd University Hospital, Casablanca, MAR; 11 Cardiology, University Clinics of Kinshasa, Kinshasa, COD

**Keywords:** abdominal pain, adrenal carcinoma, hepatic metastases, jaundice, nonfunctional adrenal incidentaloma

## Abstract

Adrenocortical carcinomas are rare but aggressive tumors that are frequently discovered as incidentalomas. Secretory tumors often lead to endocrine abnormalities, namely cushingoid features, virilization, or feminization. Non-functioning tumors, on the other hand, can be completely dormant with an insidious course or cause malaise, weight loss, abdominal pain, etc. Biochemical testing must be pursued in all patients with incidentalomas to detect pheochromocytoma, excess cortisol, or aldosterone secretion. In this report, we describe the case of a 37-year-old man who was lost to follow-up for two years following diagnosis with adrenal incidentaloma. This led to delayed diagnosis of adrenal carcinoma and eventual mortality. Periodic surveillance of adrenal incidentalomas is therefore imperative for timely interception of malignant lesions.

## Introduction

Adrenal incidentalomas are clinically inconspicuous lesions that are unexpectedly discovered during diagnostic testing for an unrelated condition. Nonfunctioning tumors measuring less than 4 cm in greatest diameter with computed tomography (CT) attenuation of less than 10 Hounsfield units (HU) generally do not require intervention. Surveillance frequency can vary based on the annual growth rate and phenotypic characteristics of the mass. Incidentalomas with indeterminate features on imaging require further testing with contrast-enhanced (CE) CT, positron emission tomography (PET)-CT, or magnetic resonance imaging (MRI). Of incidentalomas, 75% are non-functioning cortical adenomas, about 14% are functional, secreting either cortisol or aldosterone, approximately 7% are pheochromocytomas, and the remainder (4%) are primary adrenal cancers or metastases to adrenal glands [1]. The discovery of an adrenal mass should prompt radiographic and biochemical testing aimed at discerning its malignant potential and secretory function.

Adrenocortical carcinoma (ACC) is exceedingly rare, with an annual incidence of 0.5-2 patients per million population [2]. Female individuals are more frequently affected. Survival rates vary considerably, ranging from 15% to 85%, depending on the stage at which the cancer is diagnosed [3]. Lesions can be either secretory or non-secretory. Non-functioning tumors are diagnosed later in the disease course due to the absence of hormonally-mediated clinical symptoms. Treatment involves surgical resection followed by adjuvant chemotherapy in certain cases. Here we discuss a case of a patient who presented with a large painful abdominal mass and jaundice secondary to metastatic ACC.

## Case presentation

A 37-year-old man presented with a painful, right-sided abdominal mass and jaundice. The patient was diagnosed with an adrenal incidentaloma two years ago when he was treated for nephrolithiasis. At that time, the adrenal mass measured 1.2 cm with an unenhanced CT attenuation of 10 HU. Biochemical testing at that time with overnight dexamethasone suppression test (DST), and plasma dehydroepiandrosterone, metanephrines, renin, and aldosterone assays were negative. Periodic CT-based surveillance was recommended but the patient was lost to follow-up for two years.

**Figure 1:**
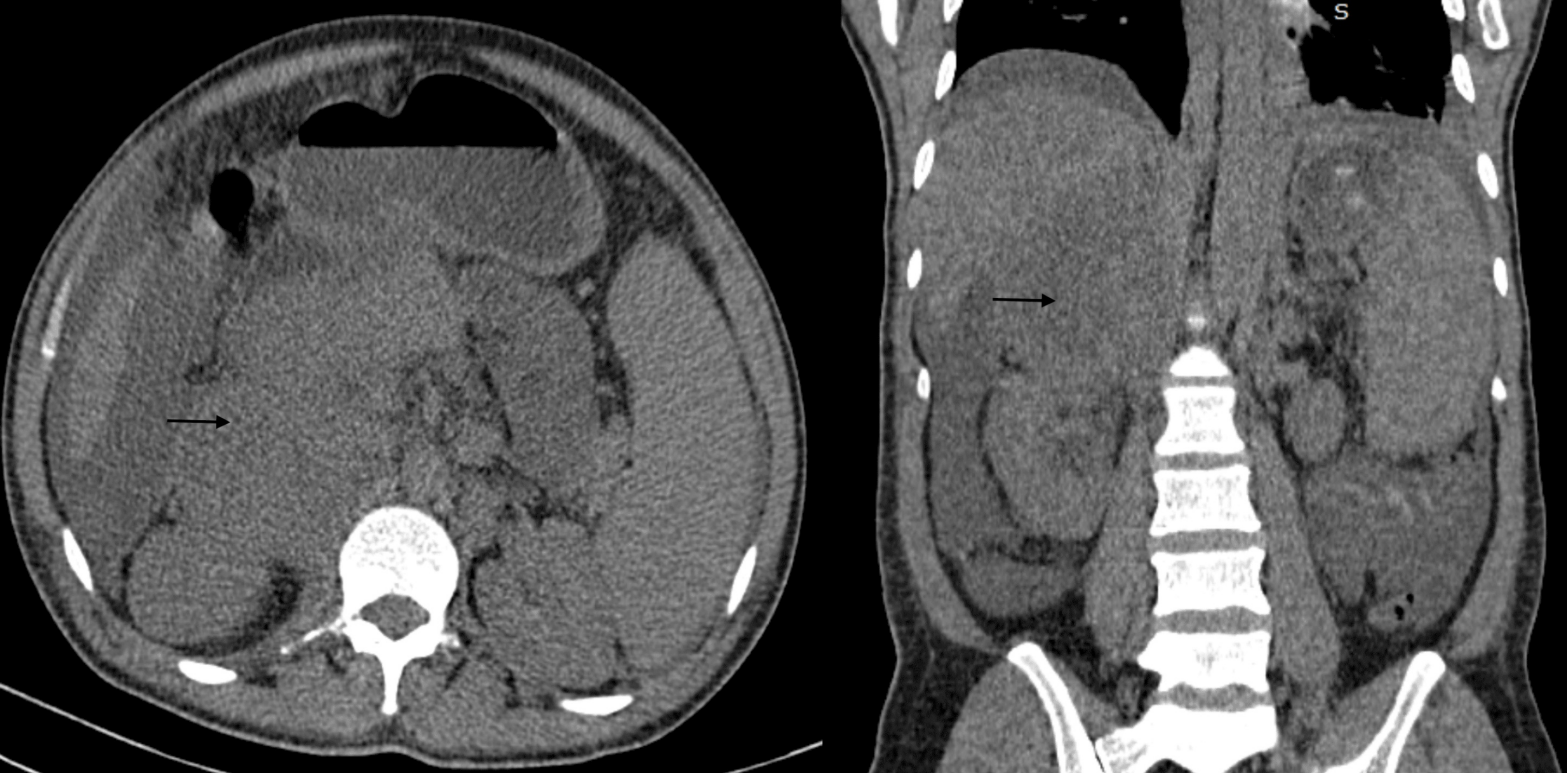
Pre-contrast CT revealing a large right adrenal mass (black arrows)

**Figure 2:**
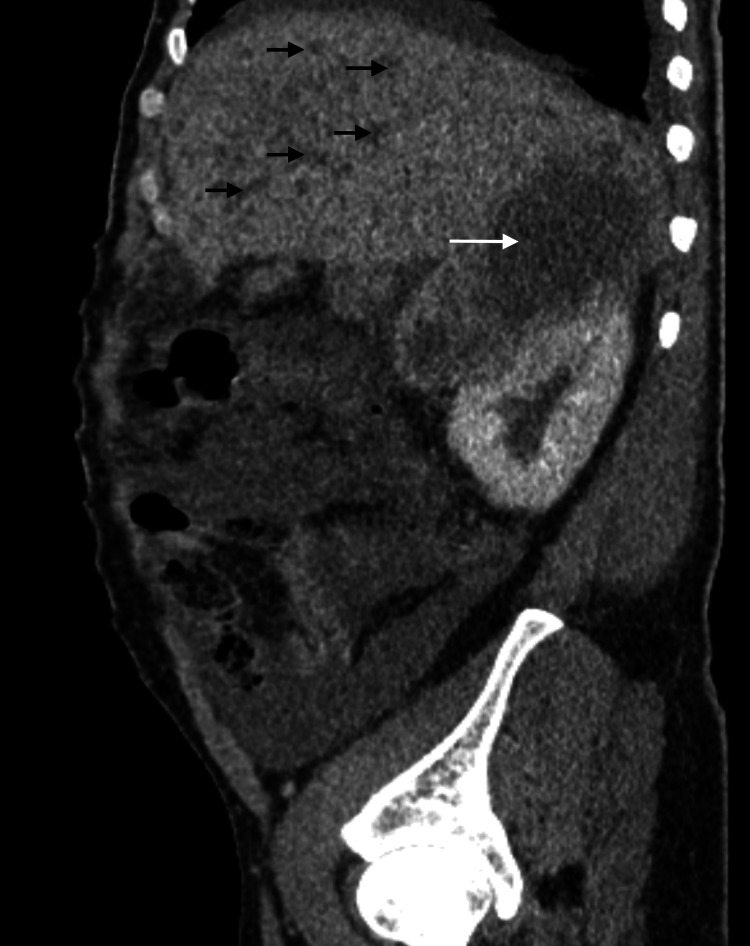
CT imaging showing multiple hypodense hepatic nodules (black arrows) and a large right adrenal mass (white arrow) measuring 14.6 x 9.4 x 9.3 cm in size

**Figure 3:**
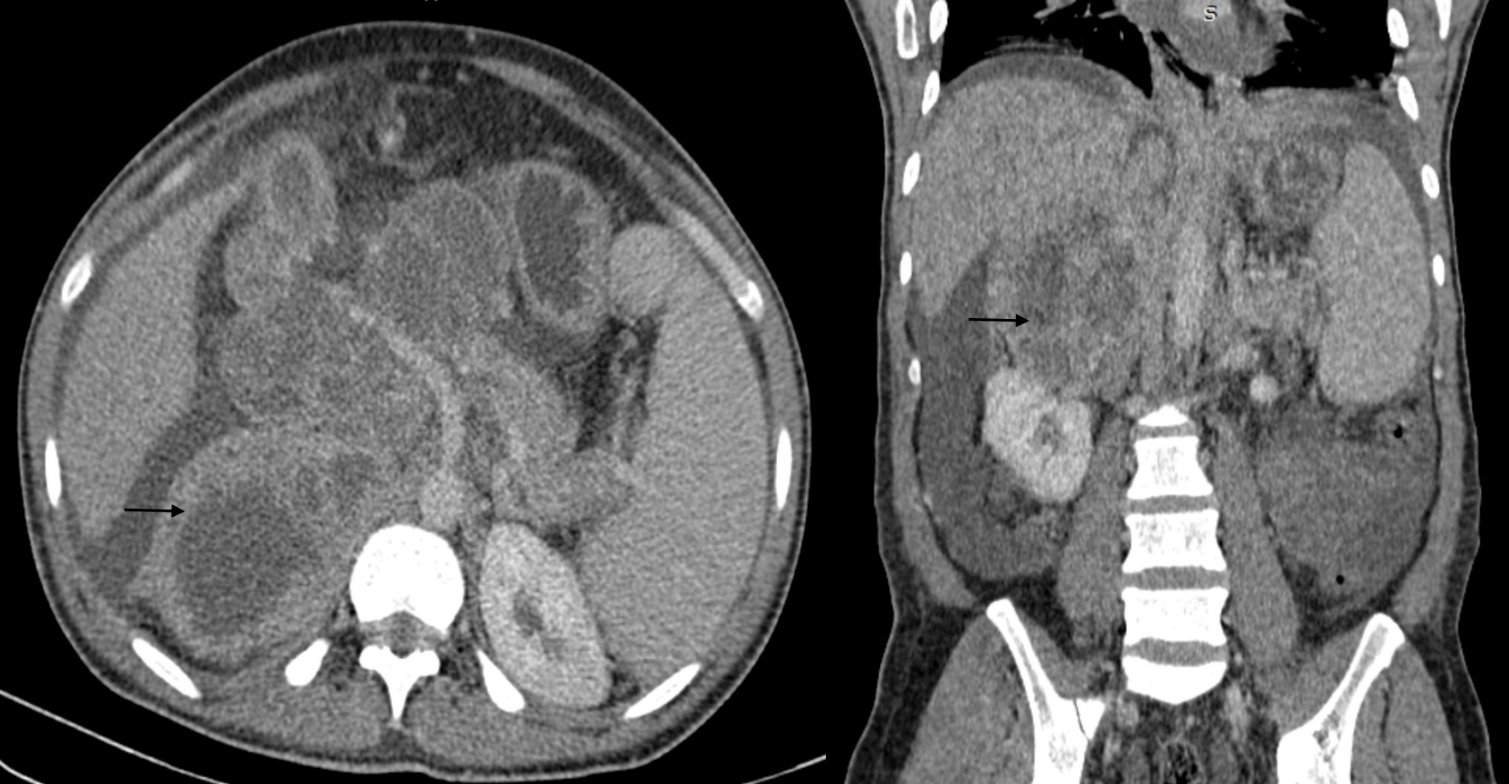
Post-contrast CT showing heterogenous enhancement of the large right adrenal mass (black arrows)

On presentation, the patient noted a two-month history of right-sided abdominal discomfort that had acutely worsened the week prior and was associated with jaundice. CT scan with contrast revealed a right adrenal mass measuring 14.6 x 9.4 x 9.3 cm in size with irregular margins and both solid and cystic components, in addition to multiple hepatic metastases. Pre and post-contrast attenuation values for the adrenal mass were 41 and 67.5 HU, respectively, with an absolute and relative washout value of 35.8% and 14.1%, respectively. These findings, collectively, were highly concerning for adrenal carcinoma (Figures 1-3).

The patient did not have clinical signs of hormonal hypersecretion. Serum bilirubin was 5.8 mg/dL. Comprehensive biochemical testing was negative with morning cortisol: 60 ng/ml (normal range: 50 - 230 ng/mL), metanephrines 46 pg/mL (normal range: 12 - 60 pg/mL), normetanephrine 75 pg/mL (normal range: 18 - 110 pg/mL), dehydroepiandrosterone: 168 mcg/mL (normal range: 45 - 270 mcg/mL), and aldosterone to renin ratio of 14 (normal < 20). Oncologic treatment of the adrenal carcinoma was deferred as the patient was hemodynamically unstable on presentation with an elevated white blood cell count of 22K. Blood cultures grew *Escherichia coli*.

The patient received aggressive fluid resuscitation and appropriate antibiotic therapy. Nonetheless, his vasopressor requirements escalated and he developed severe acute kidney injury necessitating hemodialysis. Five days into admission, he developed hypoxic respiratory failure and sustained cardiac arrest, requiring intubation and mechanical ventilation. After a prolonged stay in the intensive care unit, the patient died of multiorgan failure. 

## Discussion

ACC is a rare malignancy with an estimated annual incidence of less than two cases per million population. ACC, in various study populations, has been shown to account for up to 10% of cases of adrenal incidentalomas. The prevalence of adrenal metastases in patients with known history of primary cancer can be as high as 20% [4]. Malignancies likely to metastasize to adrenal glands include melanoma, and lung, gastrointestinal, and renal cell carcinomas. Symptomatology of an incidentaloma varies based on tumor size and functional status. Current literature states that incidentalomas greater than 4 cm in diameter are associated with an increased risk for cancer. The risk for adrenal cancer is commensurate with tumor size. Lesions less than 4 cm and between 4 to 6 cm have a low (<2%) to moderate (6%) risk for malignancy respectively. Tumors measuring 6 cm or above have a markedly increased malignancy potential (> 25%). It is important to take into account three-dimensional measurements of an incidentaloma as two-dimensional assessments often underestimate the size and eventual malignancy risk. Another caveat is the consideration of the patient’s age for estimation of cancer risk as benign incidentalomas are infrequent in patients aged less than 40 years. Concern for malignancy exists in this subset even in the presence of small incidentalomas (<4 cm) [4,5]. Close surveillance is therefore warranted in these cases.

Our patient had initially presented with a small incidentaloma and then was lost to follow-up for two years. The tumor size in our case was larger than the mean tumor size reported in current literature as adrenal carcinomas greater than 12 cm are uncommon. Functioning masses can result in Cushing syndrome, virilization, or feminization depending on the type of hormone produced [6]. Non-functioning tumors can either cause no symptoms or give rise to non-specific symptoms like malaise, abdominal discomfort, weight loss, etc. Our patient had a non-functioning tumor that led to abdominal pain and jaundice due to its large size and the presence of hepatic metastases, respectively. The lack of clinical and biochemical evidence of hormonal excess delayed our patient’s diagnosis until the occurrence of hepatic metastases. 

DST and 24-hour urinary cortisol are tested to assess for Cushing syndrome. Plasma renin and aldosterone levels should be checked in patients with hypokalemia and hypertension. Plasma or urinary metanephrines and/or catecholamines levels help rule out pheochromocytoma, and serum estrogen, androgen or dehydroepiandrosterone sulfate (DHEA-S) levels have diagnostic utility in cases with feminization or virilization. Both CT and MR imaging are useful diagnostic tools for the surveillance and phenotypic characterization of adrenal lesions [7]. The presence of irregular margins, increased vascularity, heterogeneity, and calcifications in an adrenal mass increase the likelihood of malignancy. Benign lesions typically have an attenuation of less than 10 HU on unenhanced CT. Lesions with an attenuation greater than 10 HU should be further evaluated with CE-CT, PET-CT, or MRI. Adenomas demonstrate early enhancement and washout when measured at 60-90 seconds (early enhancement) and 10-15 minutes (late enhancement) after contrast administration when compared with ACC. Absolute and relative washout of more than 60% and 40% of contrast medium respectively is also suggestive of an adenoma. Previous literature has shown that MRI-based qualitative analyses of the adrenal signal-intensity index and quantitative estimation of the adrenal-to-spleen ratio were highly accurate with a pooled sensitivity and specificity of 94% and 95% respectively. Additionally, PET-CT-based adrenal imaging can also reliably differentiate between benign and malignant lesions, with sensitivity and specificity approaching 90% in pooled analyses [8,9]. Biopsy is not recommended as it can’t reliably distinguish benign from malignant lesions, and can occasionally lead to tumor seeding if the mass is malignant. Biopsy could be performed if there is a high index of clinical suspicion regarding the presence of adrenal metastases and in cases where histopathological confirmation could impact the treatment protocol [10]. 

The presence of clinical features suggesting hormonal excess or imaging features indicative of malignancy warrant multidisciplinary involvement including an endocrinologist, surgeon, and radiologist. Functioning tumors can be managed with an adrenalectomy. Non-functioning tumors with a benign imaging phenotype can be surveilled annually for four years. During the surveillance period, if there is evidence of hormonal autonomy, annual growth rate of ≥1 cm, or a size of ≥4 cm, surgical consultation should be obtained. Surgical resection is the mainstay of treatment and is curative for localized ACC. Laparoscopic adrenalectomy can be performed by experienced surgeons to resect masses less than 6 cm [11]. Open adrenalectomy is recommended for adrenal cancers with evidence of local invasion or for masses greater than 6 cm. Local recurrence and/or distant metastases occur in more than 50% of patients. Patients with metastatic disease have limited treatment options with a five-year survival rate of 15%. Etoposide-doxorubicin-cisplatin-mitotane (EDP-M) is the standard chemotherapy regimen for advanced ACC [12,13]. Transcatheter arterial chemoembolization (TACE) and bland transarterial embolization are deemed effective options for the treatment of ACC-associated hepatic metastases [14-16]. Our patient unfortunately developed multiorgan failure secondary to septic shock, precluding him from undergoing surgical resection, adjuvant chemotherapy, and transarterial chemoembolization of liver metastases.

## Conclusions

This case highlights the importance of close surveillance of adrenal incidentalomas. Comprehensive hormonal workup must be pursued. Size is a strong predictor of malignant potential and a reliable risk stratification tool. Surgical excision yields excellent outcomes in localized ACC; therefore, early detection is important. Advanced ACCs require adjuvant chemotherapy. Transarterial embolization is a new, useful therapeutic modality for treating hepatic metastases.
